# The Incidence and Treatment of Hernia in Young Children.—II

**Published:** 1903-07-11

**Authors:** W. McAdam Eccles

**Affiliations:** Assistant Surgeon St. Bartholomew's Hospital, late Assistant Surgeon City of London Truss Society, and West London Hospital, etc.


### HOSPITAL CLINICS AND MEDICAL PROGRESS.

### THE INCIDENCE AND TREATMENT OF HERNIA IN YOUNG CHILDREN.—II.

By W. McAdam Eccles, M.S.Lond., F.R.C.S.Eng. Assistant Surgeon St. Bartholomew's Hospital, late Assistant Surgeon City of London Truss Society, and West London Hospital, etc.

In a former paper was discussed the incidence of hernia in children, much stress being laid on the fact that while there is frequently a congenital condition present predisposing to hernia, yet it is nearly always requisite that there should be a marked factor of an acquired nature in order to bring about the actual formation of a hernia consisting of a sac and its contents. In the majority of instances these two causes resolve themselves into the congenital processus vaginalis and the acquired increased abdominal pressure the outcome of intestinal distension. It follows, therefore, that the rational treatment of hernia in childhood must be the removal of these two causes. In many instances a proper dieting will obviate the formation of the intestinal gas which induces the distension. The obliteration of the sac is another matter, and it should be borne in mind that there is a natural tendency for the closure of the processus vaginalis provided that it is not kept patent by the descent into it of viscera from the interior of the abdomen. The application of a proper truss, and its continuous use for at least three years may, and in a large number of cases does, effectually prevent viscera from passing into the sac, and the efforts of nature can permanently close the process of peritoneum. This method of treatment was briefly dealt with in the preceding paper. It now remains to consider the other means whereby the processus vaginalis can be removed. This is done by the exposure of the neck of the protrusion and its obliteration by ligature. I shall discuss the question under the following headings:—(1) the indications for operation; (2) the procedure of operation; (3) the results of operation.

### 1. The Indications for Operation.

A very large number of considerations come into the question as to whether an operation should be undertaken for the relief of hernia, and with the object of its permanent cure.

*Age.*—Most surgeons are agreed that it is not well to undertake an operation in the very young, that is within the first few months of life. I consider that, unless operation is urgently called for say on account of strangulation, which by the way is comparatively rare in infancy and as a rule is easily overcome by the employment of taxis, it is well to wait at least until the early stages of the first dentition are well passed. It is no doubt true that at the present day with anæsthetics well given, with avoidance of shock, with rapidity of operating, and with rigid asepsis obtained and maintained, there is but little risk in operating in quite the early days of life, but the benefit derived is perhaps hardly commensurate with the risk even so slight as it is. There is no doubt that should the careful wearing of a suitable truss not have brought about the obvious disappearance of a hernia by the time that the child has reached the age of three, it is in most instances well not only to advocate but to urge operation with a view to a permanent cure.

*Condition of Hernia.*—This is again a very important matter in connection with the question of operation. Any hernia which contains irreducible contents, whether in whole or in part, urgently calls for operation, although in certain cases of inguinal protrusion the operator must be prepared for considerable difficulty in dealing with the condition should it turn out to be one of those not so very uncommon congenital cæcal herniæ. Umbilical herniæ which have not disappeared before the child has reached the age of three are most suitable for operative treatment.

*Condition of Parts around the Hernia.*—In connection with this point is the not infrequent association of imperfect descent of the testis with a patent processus vaginalis, and a protrusion of viscera into the pouch. Speaking generally it may be said that there is very little likelihood of a natural cure in such cases, and that operation is clearly indicated for two reasons—first, the cure of the hernia, and secondly, the transplantation of the testis.

*Points in connection with the Truss.*—If it is impossible to obtain a suitable truss, if a truss causes much inconvenience from a want of proper care in its adjustment, if a truss is not efficient, or if a truss entails too great an expense, then an operation may be indicated and may remove all the troubles that are sometimes associated with truss treatment.

*After-life of the Patient.*—In all cases where it is destined that the boy should enter one of the public services, it is well that operation should be undertaken before puberty, warning always being given that should the operation not prove a success, the boy will be debarred from passing into the service.

Given then a hernia, whether umbilical or inguinal, in a child which has reached the age of three years, and in which truss treatment has been conscientiously carried out, and all predisposing causes of hernia, except heredity, removed, it is undoubtedly advisable to urge that an operation should be undertaken.

### 2. The Procedure of Operation.

That success depends to a very large extent on the manner in which an operation for hernia is performed is almost a truism. There are at least three essential stages in the operative procedure. These are the preparation for the operation the performance of the operation and the after-treatment.

*The preparation for the operation* is of extreme importance. It comprises the attainment of asepsis, and of a condition of the general attitude of the child that will be conducive to a proper after-result. Asepsis is absolutely needful for a satisfactory termination. It is somewhat difficult to secure in a child. Not only are the ordinary obstacles to be overcome, but there are the special ones in the matter of the excreta, the urine and fæces, and the extremely sensitive skin of many children.

It is because of this latter point that great care must be taken to avoid a too energetic cleansing of the skin, for if this is done there will be a liability for so much irritation to result that the very object desired will be lost, excoriation and sepsis being induced. I consider that the best means to bring about workable asepsis are as follows:—Supposing that the operation is to be performed on the morning of a Wednesday, the child should have a hot bath on the preceding Monday evening, and after the bath the skin of the operation area and for some distance around should be rubbed over with ether, and afterwards with a watery solution of biniodide of mercury, 1–2000. Then the parts should be covered with a dressing of the double cyanide of mercury and zinc gauze, or of biniodide of mercury gauze, the best for the purpose being that prepared by Seabury and Johnson, which is beautifully soft, and very little irritating. The whole of the same process should be again carried out on the Tuesday evening, and the dressing left applied until the time of the operation.

I further consider it is by far the best plan that the child should be removed from the proximity of its parents, and that it should be kept in bed for the day before the operation under the care of comparative strangers, for in this way the child does not fret after parents and friends, is more likely to remain quiet after the operation, and is decidedly more under control. On the morning of the operation nothing whatever should be given by mouth, provided that the operation is undertaken early, say at nine o'clock. The bowels should be opened by a mild aperient given the morning before the operation and no enema should be administered.

*The Performance of the Operation.*—In the majority of instances the simpler the details of the operation the better. A general anæsthetic should always be given, and usually chloroform should be selected, and in the hands of a competent anæsthetist there should be no danger and no inconvenience. Again rigid asepsis in all matters concerned should be observed.

In the case of an umbilical hernia an incision should be made laterally half round the umbilicus, and the flap of skin thus formed raised with the scar of the navel, and the peritoneal protrusion exposed. This should be cut away flush with the parietal peritoneum, and the edges of the wound in the serous membrane brought together with fine silk sutures. The edges of the two recti muscles are then to be laid bare, by their sheaths being cut into vertically along the inner border. The two raw edges of the posterior borders of the rectus sheath are now united by the finest silk sutures, then the two muscular edges are similarly brought together, and finally the anterior edges of the rectus sheaths in like manner. After this the skin and subcutaneous tissue are sutured either with silk or fine silkworm gut. It will thus be seen that there are four layers of buried sutures and one layer of superficial stitches. The whole wound is then dressed by a gauze and collodion dressing, and a bandage applied to prevent the child from disturbing the parts. The superficial stitches are removed at the end of a week, and the scar redressed with gauze and collodion, and this left on for another week, at the end of which time the child may resume its normal conditions.

For an inguinal hernia the procedure should be equally simple. An incision is to be made well away from the scrotum, parallel with the inner half of Poupart's ligament. This on being deepened exposes the aponeurosis of the external oblique muscle. The fibres of this should be separated in the line of the incision, but it is not usually necessary to entirely open up the superficial abdominal ring. The canal is now exposed, and the ilio-inguinal nerve will be seen running down in front of the sac. This nerve should be preserved. The neck of the sac is then carefully dissected free from the cord or round ligament behind it—a matter of considerable difficulty in many cases. When the site of the deep abdominal ring has been reached, some extra-peritoneal fat will be found adherent to the neck of the sac about its mouth, and it is important that care should be here taken not to implicate the urinary bladder which may be lying very close to the inner side. The sac is opened and, if there are no contents, it is transfixed flush with the parietal peritoneum by a needle carrying fine silk and tied. The neck is then cut through below the ligature and the rest of the sac left undisturbed.

The aponeurosis of the external oblique should now be sutured, no attempt being made to fasten the arching fibres of the internal oblique and the transversais to Poupart's ligament, as would be done in a typical Bassini's operation. The skin and the subcutaneous tissue are then sutured with fine silk or silkworm gut, and a gauze and collodion dressing applied. The after-treatment is the same as that for the umbilical hernia. It is not necessary to order a truss to be worn after either operation.

### 3. The After-Results.

As a rule these leave nothing to be desired, provided that asepsis has been maintained and all predisposing causes of hernia have been removed prior to the operation. I have seen instances of recurrence of the protrusion in children who were not the subjects of robust health, and in whom there was a marked heredity tendency to hernia, and in some in whom the diet and other matters were hopelessly neglected subsequent to the operation.

To sum up, it may be said that the incidence of hernia in children while partly dependent upon a congenital predisposition is also due to acquired causes. That many cases of hernia in children are cured by the efforts of nature alone, or of nature helped by the control of the hernia by the application of a proper form of truss. That where nature fails to bring about a disappearance of the protrusion operation is indicated and that where it is carried out aseptically and with due attention to details, a permanent cure is the result.

